# Anaphylactic shock due to patent blue: case report and review of literature

**DOI:** 10.11604/pamj.2018.31.7.15576

**Published:** 2018-09-04

**Authors:** Asma Korbi, Amel Khaskhoussy, Ons Cherif, Ahmed Hajji, Imen Gaddab, Amel Chaabene, Mouna Gara, Fathi Jbeli, Lotfi Grati, Med Salah Rhim, Raja Faleha

**Affiliations:** 1 Service de Gynéco-Obstétrique Monastir CMNM 5000 Monastir, Tunisia; 2 Service de Pharmacologie Clinique, CHU Fattouma Bourguiba Monastir, 5000 Monastir, Tunisia; 3 Département d’Anesthésie Réanimation Monastir CMNM 5000 Monastir, Tunisia

**Keywords:** Breast cancer, sentinel lymph node, patent blue, allergic reaction, anaphylactic shock

## Abstract

Intraoperative search for the sentinal node using patent blue is considered a non risk procedure. We emphasize the highly exceptional nature of this adverse effect previously observed in other disciplines using this coloring agent. We present a case of allergic reaction to patent blue in a patient who underwent left mastectomy with sentinel lymph node. About 25 min after the dye injection, the patient developed increased heart frequency and allergic skin reaction. The patient was treated successfully with decreased inspired fraction of inhaled anesthetic and fluid replacement. The patient recovered uneventfully and was discharged from the PACU 3h after the end of surgery without skin changes and was discharged from hospital on the morning after surgery. Allergic reactions with the use of patent blue are far superior to the hypersensitivity reactions seen with anesthetic and adjuvant drugs. Therefore, the anesthesiologist must be aware of cardiovascular instability associated with skin changes during the use of patent blue, for early diagnosis and appropriate treatment of this hypersensitivity reaction to this dye. Skin tests done later confirmed allergy to patent blue dye; the tests induced a small syndrome reaction. Surgical personnel who use patent blue dye should be made aware of the risk of allergic reactions, sometimes severe, to this dye.

## Introduction

Identification of Sentinel lymph node in breast cancer has become a standard in the management of small lesions. Biopsy for early breast cancer surgical treatment has been widely used as part of routine protocol. In most cases, it prevents total lymphadenectomy [1]. Patent blue and isosulfan blue (2, 5-isomer of blue sulphate) are the two main dyes currently used for the identification of sentinel lymph nodes in breast cancer. This dye may be responsible for allergic reactions including anaphylactic shock [2]. The development of the sentinel lymph node technique in breast cancer confronts us more and more with this potentially serious problem. There are reports of hypersensitivity reactions mediated by IgE to blue dye (average incidence of 1.8% (0.1% to 2.8%) [3]. In some cases, these reactions can be severe and lead to serious hemodynamic effects requiring vasoactive drugs. This frequency is higher than the hypersensitivity reactions seen during anesthesia, which is around 0.01% to 0.02%. Another effect seen with the use of patent blue are pulse oximetry changes because it interferes with the wavelength reading used to measure the oxyhemoglobin [3]. The objective of this study is to present a case of intraoperative allergic reaction after subdermal peri-areolar injection of patent blue dye.

## Patient and observation

**Figure 1:**
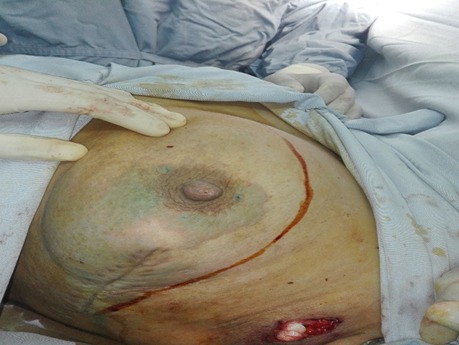
Acute blue urticaria due to patent blue beginning on the per areolar area, Korbi et al, 2018, Monastir,Tunisia

**Figure 2:**
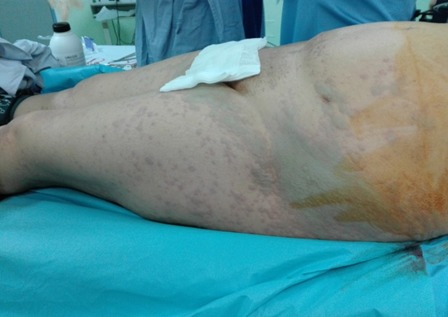
Acute blue urticaria due to patent blue spreads to the whole body, Korbi et al, 2018, Monastir, Tunisia

**Figure 3:**
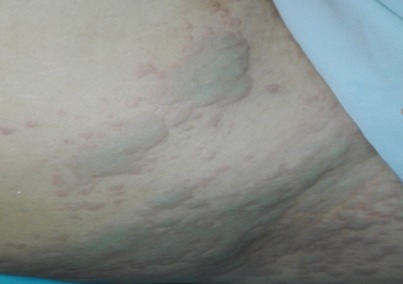
Acute blue urticaria due to patent blue, Korbi et al, 2018, Monastir,Tunisia

Female patient, 45 years old, referred for left mastectomy with sentinel lymph node resection. There was no other comorbidities; she denied smoking and allergies to medicines, foods and latex. Preoperative tests, such as cardiac examination, blood count, coagulation, biochemistry (urea, glucose, creatinine, AST, ALT) and urinalysis were normal. The patient was not premedicated. At the operating room, intra venous infusion was performed at the right upper limb with 20G Teflon catheter. Basic monitoring with cardioscope, pulse oximeter, and non invasive blood pressure showed normal sinus rhythm, heart rate (HR) of 78 bpm, oxygen saturation (SpO2) of 98% and blood pressure of 120 × 70 mmHg. After preoxygenation with 100% O2 via face mask and administration of cefazolin(2 g), induction of anesthesia was achieved with fentanyl (250 g), propofol (150 mg) and rocuronium (50 mg). Tracheal intubation was performed with a 7.5 mm cuffed tube and basic monitoring complemented with capnography; controlled mechanical ventilation with 570 mL tidal volume and 12 ipm respiratory rate. At that point, cardiorespiratory parameters evidenced normal sinus rhythm, HR 84 bpm, SpO2 100%, ETCO2 30, and PA 110 × 75 mmHg. Maintenance of anesthesia was achieved with sevoflurane (2---2.5%) and O2/N2O (50/50%). Immediately before the incision, a subdermal per areolarinjection of 2.5% patent blue (2 mL) was performed. About 25 min after the blue dye injection, increased HR (110 bpm) and allergic skin reaction occurred without changes in blood pressure, SpO2, and capnography. Ringer's lactate (400 mL) was administered and end-tidal sevoflurane concentration decreased to 1.5%. About 10 min after volume replacement and a decrease in the fraction of inspired halogenated, blood pressure was 100 × 60 mmHg and HR 85 bpm, without changes in the remaining parameters. The anesthetic-surgical procedure went through without complications. Dipyrone (2 g) was used for postoperative analgesia. The duration of surgery was 50 min. At the end of the procedure, atropine (1 mg) and neostigmine (2 mg) were used for neuromuscular blockade reversal. After surgical field removal, the presence of numerous urticaria-like plates (bluish) was observed mainly in the face, neck, upper limbs, and thorax (Figure 1, Figure 2, Figure 3). Hydrocortisone (500 mg) was administered. The patient quickly awakened from anesthesia and was extubated in the operating room. She was conscious, free of pain and with cardiovascular and respiratory stability (blood pressure: 120 × 80 mmHg, HR: 78 bpm, and SpO2: 98%). The patient was taken to the post-anesthesia care unit (PACU). After 60 min, the patient had no skin changes and was discharged from PACU 180 min after surgery. The patient was discharged from hospital the morning after surgery without complications. Patient gave her informed written consent for publication.

## Discussion

Axillary dissection remains a fundamental prognostic interest in breast cancer [2] but its disadvantages are well known. Several researches to avoid it are under study. The identification of prognostic factors on the tumor is still lacking power. The technique of identification of the sentinel lymph node could prevent a large number of women the consequences of extensive axillary dissection. Two methods of identifying sentinel lymph nodes have been proposed: the isotopic method and the “patent blue” dye method. This sentinel lymph node technique was proposed by Giuliano in 1994 [4]. To date, we have found few anaphylactic accident publication during sentinel lymph node research.Our observation concerned an allergic and anaphylactic event. It had no serious consequences. The imputability of patented blue is plausible. Radiologists, angiologists and dermatologists had already published some observations of “patent blue” accidents described as “green urticaria”. Anaphylactic shocks and urticarial reactions have been described. In general, anaphylactic shock occurs within ten minutes of the intravenous injection of the causative agent. This delay is slightly longer in case of intradermal injection [1]: 15 minutes minimum up to 45 minutes for a team [3]. In our case, the delay was 25 minutes. This time corresponds to the systemic diffusion time of the product. Albo *et al* even describe two cases in his series of seven anaphylactic shocks that presented a second episode a few hours after the first reaction [1]. Our patient did not have a history of allergic reaction .In practice, we never inject blue in patients with known allergic history. This attitude based on a precautionary principle can be discussed since King *et al* have not found a relationship between allergic reactions to blue and a history of allergy to other substances [5]. Since 2001, several articles have described allergic phenomena related to this blue dye and generally have an incidence of less than 1% [1]. The incidence is fairly variably reported and it must be recognized that it is not known exactly. The incidence of shock ranged from 0% in 416 patients [6] to 1.1% in 369 patients [1] and the average we can calculate is 0, 25% for anaphylactic shock. These are studies in which the authors mainly use isosulfan blue, much more rarely patent blue [7]. For patented blue, Léong *et al* . have reported an incidence of 0.6 to 2.5% of allergic reactions in the literature [8], but without specifying the percentage of anaphylactic shock. Raut *et al* . studied the impact of anti-allergic prophylaxis with corticosteroids and histamine receptor antagonists in a study of 1013 patients [9]. This author used a 5ml injection of 1% isosulfan blue. Before initiation of prophylaxis, he reported an incidence of 1.1% anaphylactic shock for 639 patients and 0.7% after anti-allergic prophylaxis. This prophylaxis did not decrease the incidence of allergic reactions (p = 0.30), but it appears to decrease the severity of the reaction.

These accidents were observed during pedal lymphography (about thirty cases were reported). Diagnostic tests in lymphoedema for evidence of a lymphatic etiology would have caused accidents identical to ours. The accident is rare [10] reports 3,000 lymphographies without incident. The “patent blue” would be the responsible. For a long time the local anesthetic injected at the same time was incriminated, but Barber [2] observed a case without the use of local anesthesia. There would be no 100% reliable screening test [2]. The dose would not intervene. The blue color of the papules is suggestive of the imputability of the dye. This type of accident must be known to avoid therapeutic errors. Isotopic tracking techniques [11] are much more expensive and more complex to implement. The dyeing technique could sometimes be associated with isotopes [12]. The simplicity and the good agreement observed by a large number of authors of the “patent blue” method [13] make it keep all its interest. The anaphylactic shock due to patent blue is probably rare, but it must be suspected rapidly when hemodynamic instability appears after injection of blue [14]. In addition to the resuscitation procedures, specimens should be taken within 30 to 60 minutes: histamine, tryptase and specific IgE assay in a dry tube (7 mL) and an EDTA tube (7 mL) and sent to the local laboratory within two hours or refrigerator storage at 4°C for 12 hours maximum. A systematic allergological survey in case of anaphylactic shock is mandatory [3, 15, 16]. It should not be done until at least four to six weeks after the event. It allows for skin tests that will confirm the responsibility of blue. Reporting to the regional pharmacovigilance center is imperative. There is currently no way to determine the risk of a reaction to vital blue dyes. Some surgical centers have attempted to use only isotopes for the identification of sentinel lymph nodes instead of a combination of isotope and patent blue [17]. Nevertheless, in about 10% of cases, the sentinel lymph node was detected by the dye blue, whereas it was not labeled by the isotope [18]. According to Raut *et al* , [19] preoperative premedication with antiallergic drugs would not prevent severe anaphylactic reactions with isosulfan blue, but could limit their severity.

## Conclusion

In perioperative reactions in surgery, the potential involvement of vital blue dyesmust not be forgotten. Serum tryptase should be determined during any perioperative reaction, ideally within 30 minutes to six hours. The allergic reaction, possibly incriminable substances and the temporal relationship of their administration with the onset of symptoms must be thoroughly documented. If a reaction to vital dyes is suspected, the absorption and prolonged half-life of these substances should be considered and extended monitoring should be provided. Thereafter, patients should be referred for allergic assessment. The allergy assessment will be based on perioperative documentation, the accountability of the substances administered, serum tryptase levels and skin tests.

## Competing interests

The authors declare no competing interest.
